# Skin Improvement Effects of Ultrasound-Enzyme-Treated Collagen Peptide Extracts from Flatfish (*Paralichthys olivaceus*) Skin in an In Vitro Model

**DOI:** 10.3390/ijms25179300

**Published:** 2024-08-27

**Authors:** Su-Jin Eom, Jae-Hoon Kim, A-Reum Ryu, Heejin Park, Jae-Hoon Lee, Jung-Hyun Park, Nam-Hyouck Lee, Saerom Lee, Tae-Gyu Lim, Min-Cheol Kang, Kyung-Mo Song

**Affiliations:** 1 Food Processing Research Group, Korea Food Research Institute, Wanju-gun 55365, Republic of Korea; eom.su-jin@kfri.re.kr (S.-J.E.); k.jaehoon@kfri.re.kr (J.-H.K.); areumryu@kfri.re.kr (A.-R.R.); parkhj@kfri.re.kr (H.P.); jokko77@gmail.com (J.-H.L.); lnh3fc@gmail.com (N.-H.L.); 2 Department of Food Science & Biotechnology, Sejong University, Seoul 05006, Republic of Korea; tglim@sejong.ac.kr; 3 Department of Food Science and Technology, Jeonbuk National University, Jeonju 54896, Republic of Korea; 4 Infrastructure Support Team, Korea Food Research Institute, Wanju-gun 55365, Republic of Korea; pjh@kfri.re.kr; 5 3FC Corporation, Wanju-gun 55365, Republic of Korea; 3fclsr@gmail.com; 6 Carbohydrate Bioproduct Research Center, Sejong University, Seoul 05006, Republic of Korea; 7 Department of Food Science & Biotechnology, Sungshin Women’s University, Seoul 01133, Republic of Korea

**Keywords:** collagen, peptide, ultrasound, enzyme, skin functionality

## Abstract

Collagen is considered to be an intercellular adhesive that prevents tissue stretching or damage. It is widely utilized in cosmetic skin solutions, drug delivery, vitreous substitutions, 3D cell cultures, and surgery. In this study, we report the development of a green technology for manufacturing collagen peptides from flatfish skin using ultrasound and enzymatic treatment and a subsequent assessment on skin functionality. First, flatfish skin was extracted using ultrasound in distilled water (DW) for 6 h at 80 °C. Molecular weight analysis via high-performance liquid chromatography (HPLC) after treatment with industrial enzymes (alcalase, papain, protamex, and flavourzyme) showed that the smallest molecular weight (3.56 kDa) was achieved by adding papain (0.5% for 2 h). To determine functionality based on peptide molecular weight, two fractions of 1100 Da and 468 Da were obtained through separation using Sephadex™ G-10. We evaluated the effects of these peptides on protection against oxidative stress in human keratinocytes (HaCaT) cells, inhibition of MMP-1 expression in human dermal fibroblast (HDF) cells, reduction in melanin content, and the inhibition of tyrosinase enzyme activity in murine melanoma (B16F10) cells. These results demonstrate that the isolated low-molecular-weight peptides exhibit superior skin anti-oxidant, anti-wrinkle, and whitening properties.

## 1. Introduction

In recent years, “sustainable development” has become a cornerstone of green economic initiatives. Owing to the increasing environmental and economic problems worldwide, there is an urgent need to adopt methods that use eco-friendly resources. The European Commission’s “Blue Growth” strategy supports sustainable growth in marine ecosystems. The fish industry generates byproducts that account for more than half the weight of fresh fish, and over 20 million tons of marine waste (fins, heads, skin, and intestines) are produced annually. To address this issue, several studies have been focused on the recovery of valuable components from marine waste, including collagen, gelatin, physiologically active peptides, amino acids, oils, pigments, and chitin [1,2].

The word “collagen” comes from the Greek term “Kolla,” which means glue. Collagen serves as an intercellular adhesive, preventing tissue stretching or damage. Sheep intestines were first used as a suture material in 1881 for the use of collagen as a biomaterial. The first collagen-based bone transplant was approved in 1993, and collagen was regarded to be “generally recognized as safe” (GRAS) by the US Food and Drug Administration (FDA) [3]. It has since been used in cosmeceuticals, drug delivery, vitreous substitutions, 3D cell cultures, and surgery, and its global market growth is expected to reach USD 6.63 billion by 2025 [4].

Marine collagen can be obtained not only from vertebrates but also from invertebrates, which constitute the majority of the animal kingdom and are abundant in terms of collagen. However, their complex structures and low solubility pose challenges for collagen extraction. In contrast, vertebrates such as fish, eels, turtles, and salamanders offer advantages in terms of their easy extraction and high purity [5].

Mechanical treatments, such as soaking, peeling, dehairing, and cutting, as well as chemical pretreatments with acids and alkalis, are required for collagen extraction. Following these steps, collagen is extracted via acid and alkali hydrolysis, salt solubilization, and enzymatic hydrolysis [6]. These methods are conventionally used for collagen extraction; however, a green technology, the ultrasound-assisted extraction method, has also been used for collagen extraction from marine sources. Ultrasonic waves cause cavitation, creating cavitation bubbles that can increase the extraction yield without altering the physicochemical properties of collagen [7]. To extract insoluble natural collagen, heat treatments sever the hydrogen and covalent bonds within collagen, destabilizing its triple-helical structure. This process produces partially hydrolyzed water-soluble gelatin [8], which is hydrolyzed by enzymes, salts, and acids, resulting in the breakdown of collagen peptides (collagen hydrolysates) [9].

Studies using animal models and clinical trials have reported the beneficial effects of collagen peptides, including enhanced wound healing, the prevention and treatment of osteoporosis and osteoarthritis, reduced risk of cardiovascular diseases, as well as anti-aging, anti-inflammatory, anti-oxidant, and anti-tumor effects [10].

In addition to natural peptides, studies on the pharmaceutical activity of synthetic peptides in the skin are ongoing. Although synthetic peptides exhibit high activity, their absorption rates and stabilities are relatively low. To address this, they are often modified by attaching fatty acids, such as palmitate, acetyl groups, or metal ions, such as copper or manganese [11]. Although the clinical benefits of polypeptides have been demonstrated, understanding their mechanisms of action and their large-scale screening, purification, and production remains challenging. In addition, peptides derived from natural sources often suffer from low yields or complex extraction processes that make it difficult to extract them in large volumes.

A previous study described an ultrasonic mass production process to extract collagen from flatfish skin, a marine byproduct, taking advantage of sustainable development and blue growth [12]. As a follow-up to that study, we aimed to prepare collagen peptides by using an enzymatic treatment and verify their functionality related to anti-oxidant, anti-wrinkle, and whitening effects on the skin, thereby increasing the industrial utilization of eco-friendly extracted collagen peptides.

## 2. Results and Discussion

### 2.1. Ultrasound-Based Collagen Extraction from Flatfish Skin

In a previous study [12], we developed an ultrasonic mass-production system capable of processing up to 60 L of collagen extracted from flatfish skin for food industry applications. Collagen from flatfish skin was extracted under similar ultrasound conditions (extraction solvent: DW; extraction time: 6 h; and extraction temperature: 80 °C). Most marine collagen obtained from vertebrates is type I. However, type II collagen can be obtained from the cartilage of fish, skates, and sharks, whereas type IV collagen can be obtained from invertebrates, such as crustaceans and mollusks [5]. There are approximately 28 types of collagen, classified based on their binding and composition, each with different functions and applications. Type I collagen is mainly found in the skin, bones, teeth, tendons, ligaments, and vascular ligatures. Owing to its high biocompatibility with the human body, it is effective in reducing wrinkles, regenerating skin, and reversing skin aging [13].

### 2.2. Enzyme Treatment of Ultrasonicated Collagen

#### 2.2.1. Enzyme Reaction Condition

**Figure 1:**
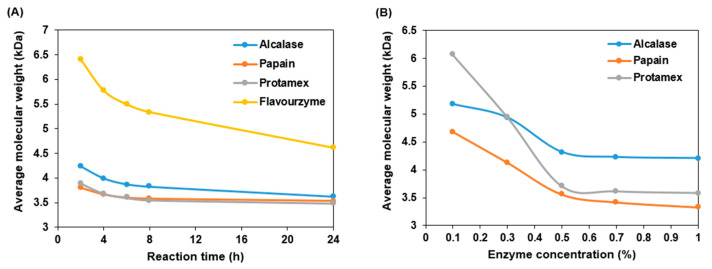
Molecular weight changes in ultrasonicated collagen according to reaction time (**A**) and concentration of enzyme (alcalase, papain, protamex, and flavourzyme) (**B**).

Four industrial proteases, namely alcalase, papain, protamex, and flavourzyme, were used to prepare the collagen peptides. To determine the optimal conditions for the enzymatic treatment, the change in molecular weight of the collagen peptides according to the reaction time and concentration of the enzyme was measured using HPLC. Figure 1A shows the molecular weights of collagen peptides at 2, 4, 6, 8, and 24 h after adding 0.5% (*w*/*w*) of each enzyme relative to the substrate. Papain, protamex, alcalase, and flavourzyme showed the fastest collagen decomposition rates. Despite a 24h enzyme reaction period, flavourzyme produced a minimum molecular weight of 4.6 kDa, which is inferior to the other enzymes. Thus, flavourzyme was excluded from subsequent experiments. Figure 1B shows the molecular weights of collagen peptides after adding 0.1, 0.3, 0.5, 0.7, and 1.0% (*w*/*w*) of each enzyme relative to the substrate, with a reaction time of 2 h. Consistent with the previous findings, collagen degradation was highest in the order of papain, protamex, and alcalase. The majority of the collagen was decomposed when the enzyme concentration was 0.5% (*w*/*w*). Therefore, subsequent experiments were conducted with 0.5% (*w*/*w*) papain-treated peptides for 2 h.

#### 2.2.2. Skin Anti-Oxidant Effect of Ultrasound-Enzyme Treated Collagen Peptide

**Figure 2:**
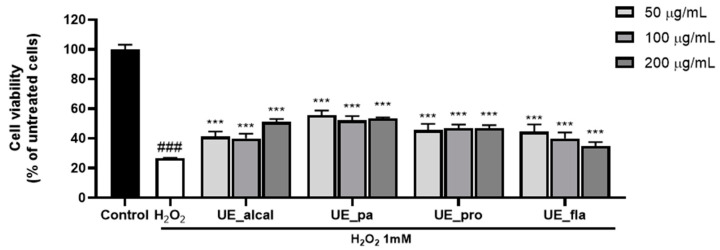
Cell viability of H_2_O_2_-treated HaCaT cells incubated with ultrasound-enzyme (0.5% alcalase, papain, protamex, and flavourzyme [*w*/*w*] for 2 h)-treated collagen. ### *p* < 0.001 compared to untreated group. *** *p* < 0.001 compared to the H_2_O_2_-treated group.

To determine the skin functionality of the ultrasound enzyme-treated collagen peptides, cell viability was measured in H_2_O_2_-treated HaCaT cells. HaCaT cells, which are human keratinocytes, are primarily used as a model to verify the protective effect of skin by assessing the degree of cell damage caused by oxidative stress [14,15]. When treated with 1 mM H_2_O_2_, the cell survival rate was reduced to 26.8% (Figure 2). However, when collagen peptides were applied at concentrations of 50–200 µg/mL, all samples exhibited a protective effect against oxidative stress. Although the differences in cell viabilities among the enzyme-treated samples were not significant, the highest cell survival rate of 55.7% was observed with papain as the enzyme.

Papain is a representative plant protease that differs from animal proteases (pepsin, pancreatin, and trypsin) as well as from microorganism-derived enzymes (alcalase, protamex, and flavourzyme). The use of animal enzymes may be restricted depending on the health problems caused by the outbreak of infectious diseases or due to religious concerns [16]. In the food industry, plant and microbial enzymes enhance flavor, nutritional value, solubility, and digestibility [17]. However, microbial enzymes require complex fermentation and purification processes, and some can cause allergic reactions [18]. Plant enzymes also have the disadvantage of being less stable than microbial enzymes; however, studies that extracted collagen from snakehead fish [19], redbelly yellowtail fusiliers [20], and chicken feet [21] have shown that papain is suitable for collagen extraction.

The correlation analysis between the molecular weight of collagen peptides and the cell survival rate under oxidative stress revealed a Pearson correlation value of −0.910 (*p* < 0.001). This indicated that as the molecular weight decreased, the cell survival rate increased significantly. In another study that confirmed the oxidative stress reduction effect of collagen peptides in HaCaT cells, the molecular weight of the enzyme-treated collagen peptides was less than 3 kDa [22]. In addition, the molecular weight of collagen peptides treated with papain in Asian sea bass skin was approximately 3 kDa, and the protective effect against oxidative stress in HaCaT cells was verified in relation to fluorescence intensity and apoptosis [23].

#### 2.2.3. Skin Anti-Wrinkle Effect of Ultrasound-Papain Treated Collagen Peptide

Skin damage is caused by oxidative stress induced by ultraviolet (UV) radiation. When skin is exposed to UV radiation, rays penetrate the epidermis and reach the dermis, causing numerous problems, such as oxidative stress, DNA damage, alterations in the cell cycle, and mutations [24]. Previous experiments have verified the functionality of collagen peptides in skin epidermis cells (HaCaT). We verified the functionality of our isolated collagen peptides in the skin dermis (human dermal fibroblasts, HDF cells) through MMP-1 expression measurements. UV exposure in skin cells increases MMP-1 expression, which breaks down dermal collagen and causes skin damage [25].

**Figure 3:**
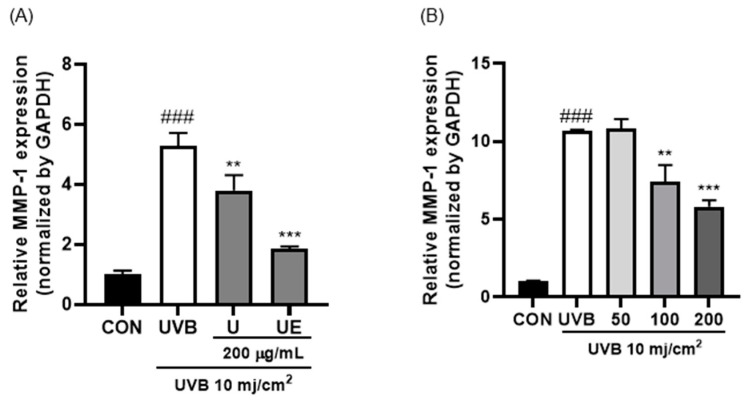
Relative MMP-1 expression (normalized to GAPDH) in UVB-induced HDF cells via ultrasound–enzyme (0.5% papain (*w*/*w*) for 2 h)-treated collagen peptide. (**A**) The effect of the presence or absence of ultrasound and (**B**) the effect of different sample concentrations. ### *p* < 0.001 compared to untreated group. ** *p* < 0.05 and *** *p* < 0.001 compared to UVB-treated group.

Figure 3 shows the MMP-1 expression induced by UVB in HDF cells using qPCR. As shown in Figure 3A, MMP-1 expression was significantly reduced two-fold when the cells were treated with ultrasound–enzyme (papain) collagen peptide (UE) compared to that with ultrasound-treated collagen (U). In addition, when UE was treated at 50 to 200 μg/mL, MMP-1 expression decreased in a concentration-dependent manner (Figure 3B).

MMPs are crucial for skin photoaging and are capable of degrading almost all the components of the extracellular matrix (ECM), including collagen, fibronectin, elastin, and proteoglycans. Specifically, MMP-1 breaks down type I and III collagen [26]. A study on the inhibitory effect of wrinkle production reported that oyster (Carsostreagias gigas) enzyme hydrolysates not only inhibit collagen degradation through the regulation of MMPs, MAPKs (mitogen-activated protein kinases), and AP-1 (Activator protein 1) expression but also affect collagen resynthesis through TGFβ-/Smad (transforming growth factor beta-/small mothers against decapentaplegic homolog) signaling pathway regulation [27]. In addition, after two weeks of collagen peptide administration to SKH-1 hairless mice and UVB irradiation, the depth of wrinkles decreased, and MMP-1, MMP-13, and reactive oxygen species were inhibited [28].

### 2.3. Isolation of Ultrasound-Papain Treated Collagen Peptide

**Figure 4:**
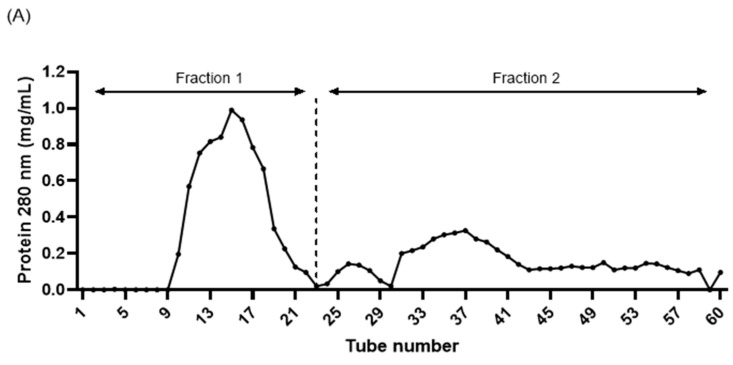

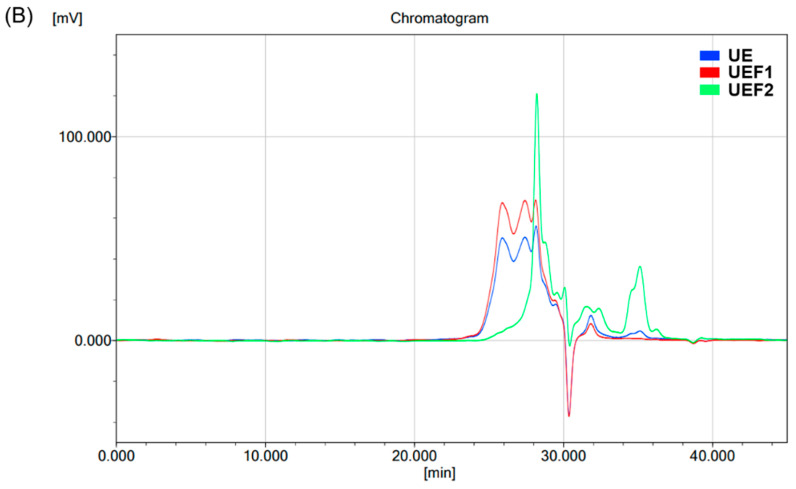
Separation of ultrasound–enzyme (0.5% papain [*w*/*w*] for 2 h)-treated collagen peptides using Sephadex G-10 (**A**), molecular weight analysis using gel permeation chromatography (**B**).

Our results demonstrated the highest skin functionality with collagen peptides treated with ultrasound–papain (UE). Next, we separated the peptides via gel filtration to determine the degree of skin functionality based on the molecular weight of the collagen peptides. As a result of the peptide separation shown in Figure 4A, 60 tubes were collected, with tubes 1–23 categorized as fraction 1 (UEF1) and tubes 24–60 categorized as fraction 2 (UEF2). To determine the differences in the molecular weights of the fractions, the protein molecular weights were analyzed using GPC. As shown in Figure 4B and Table 1, the molecular weight of UE was analyzed using KOPTRI, with a weight-average molecular weight (Mw) of 1095 Da. In addition, UPF1, which was fractionated via gel filtration, was measured to be 1100 Da, whereas UPF2 was analyzed as having the smallest molecular weight of 468 Da.

### 2.4. Skin Functionality of Ultrasound-Papain Treated Low-Molecular-Weight Collagen Peptide

#### 2.4.1. Anti-Wrinkle Effect

**Figure 5:**
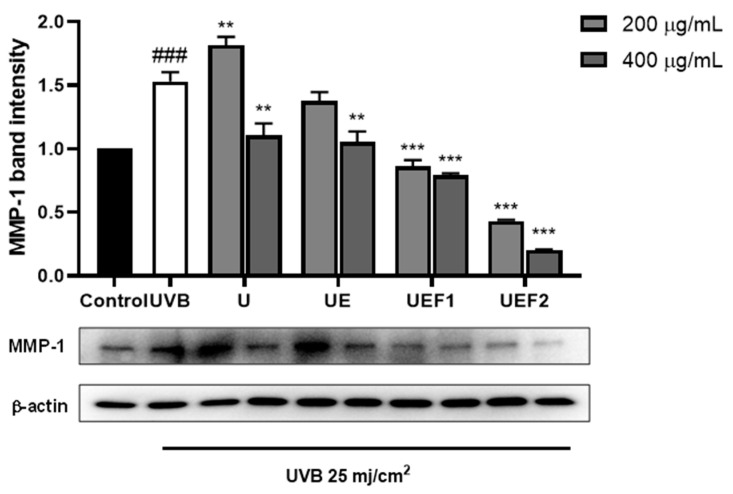
MMP-1 expression in UVB-induced HDF cells via ultrasound-treated collagen (U), ultrasound–enzyme (papain)-treated collagen peptide (UE), UE fraction 1 (UEF1), and UE fraction 2 (UEF2). ### *p* < 0.001 compared to untreated group. ** *p* < 0.05 and *** *p* < 0.001 compared to UVB treated group.

To investigate skin wrinkle reduction when using collagen peptides based on their molecular weight distribution, MMP-1 expression in UVB-irradiated HDF cells was measured via Western blot analysis. As shown in Figure 5, as the extraction and fractionation processes proceeded, MMP-1 expression decreased in accordance with sample concentration. UEF2, with the lowest molecular weight, exhibited the strongest anti-wrinkle effect.

#### 2.4.2. Whitening Effect

**Figure 6:**
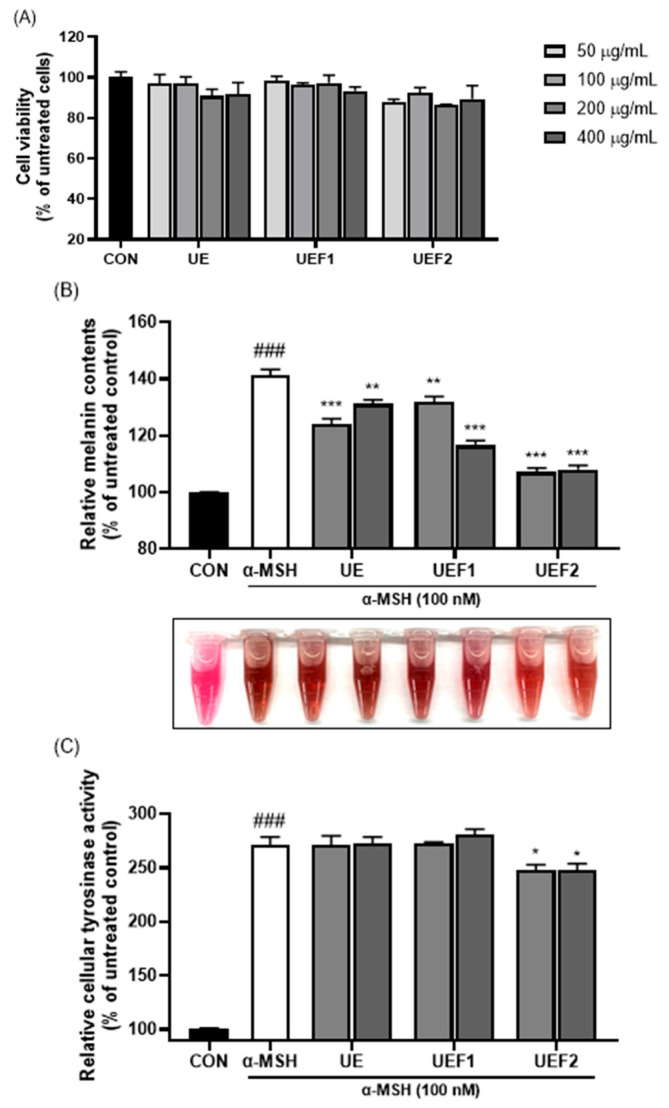
Cell viability in B16F10 cells of UEs (**A**), extracellular melanin contents in α-MSH-induced B16F10 cells of UEs (**B**), and cellular tyrosinase activity in α-MSH-induced B16F10 cells of UEs (**C**). ### *p* < 0.001 compared to untreated group. * *p* < 0.01, ** *p* < 0.05 and *** *p* < 0.001 compared to α-MSH treated group.

To assess the whitening effect of low-molecular-weight collagen peptides, melanin content and tyrosinase activity were measured in B16F10 cells. Figure 6A confirms that when the samples were treated with concentrations ranging from 50 to 400 µg/mL, the minimum cell viability was 86%, indicating the minimal cytotoxicity of collagen to B16F10 cells. Figure 6B shows the increase in melanin pigment content following treatment with α-MSH. However, the addition of UE reduced melanin production. Additionally, low-molecular-weight collagen peptides (UEF2) exhibited stronger inhibitory effects on melanin production than high-molecular-weight collagen peptides (UEF1). Tyrosinase, a factor that affects melanin synthesis, catalyzes the oxidation of L-tyrosine to L-DOPA. Subsequently, L-DOPA is oxidized to dopaquinone, which influences melanin production [29]. As shown in Figure 6C, the measurement of tyrosinase activity revealed that treatment with UEF2 inhibited enzyme activity, thereby enhancing the inhibition of melanin synthesis. Melanin biosynthesis after dopaquinone treatment involves melanocyte-inducing transcription factor (MITF), which regulates the expression of tyrosinase, tyrosinase-related protein-1 (TRP-1), and tyrosinase-related protein-2 (TRP-2) [30]. Although tyrosinase activity was somewhat lower in the collagen peptide samples, a decrease in melanogenesis may inhibit these factors, thereby lowering MITF levels.

## 3. Materials and Methods

### 3.1. Ultrasound Treatment for Collagen Extraction from Flatfish (Paralichthys olivaceus) Skin

Flatfish skin was obtained from a local fishery facility (Wanju, Jeonbuk, Republic of Korea). The skin was then cut into small pieces and the cut skin (200 g) was mixed with 800 mL of distilled water. The skin was extracted using a VCX750 (Sonics & materials inc, Newtown, CT, USA) under the following conditions: 20 kHz, 60% amplitude, 10 s/10 s on/off pulses, 80 °C, and 6 h. After extraction, the extract was centrifuged (3500× *g*, 25 min, 4 °C) to obtain the supernatant. The supernatant was filtered to remove impurities and then freeze-dried.

### 3.2. Enzyme Treatment of Ultrasound Treated Collagen

Industrial protein-hydrolyzing enzymes were purchased from Novozymes (Bagsvaerd, Denmark). Alcalase (EC 3.4.21.14) was derived from *Bacillus licheniformis* and Protamex (EC 3.4.21.63) was derived from *Bacillus* sp. Flavourzyme (EC 3.4.21.63) was derived from *Aspergillus oryzae*, and papain was derived from papaya latex (EC 3.4.22.2). After reacting for 2, 4, 6, 8, and 24 h with the addition of 0.1% to 1% enzyme to ultrasound-treated collagen, the enzymes were inactivated by heating at 100 °C for 5 min.

### 3.3. Separation of Ultrasound-Enzyme Treated Collagen

The samples were subjected to size-exclusion chromatography using a Sephadex G-10 (Cytiva, Uppsala, Sweden) gel filtration column (40 × 3 cm) [31]. The column was washed with DW, and 5 mL of the sample solution (200 mg/mL) was loaded. DW was used as the eluent at a flow rate of 2 mL/min. The fractions (10 mL) were collected in tubes using a fraction collector (QT-80FC-LCD, QiTe, Shanghai, China). The protein concentration in each fraction was measured using a BioPhotometer D30 (Eppendorf, CT, USA).

### 3.4. Analysis of Molecular Weight

HA high-performance liquid chromatography (HPLC; UltiMate 3000, Thermo Fisher Scientific, Waltham, MA, USA) was used to analyze the molecular weight distributions of the samples. The samples were filtered using a 0.45 μm syringe filter, and the samples were introduced into a column (protein KW-804; 8 mm × 300 mm, 4 μm; Shodex, Tokyo, Japan). A 50 mM sodium phosphate buffer containing 0.3 M NaCl was adopted as the mobile phase, the injection volume was 10 μL, the flow rate was 1 mL/min, and the ultraviolet (UV) wavelength was 220 nm. Standard samples consisting of alcohol dehydrogenase (150,000 Da), bovine serum albumin (66,000 Da), carbonic anhydrase (29,000 Da), cytochrome c (12,400 Da), aprotinin (6500 Da), and cyanocobalamin (1350 Da) were loaded onto the column [32]. The molecular weight of the fractionated collagen peptide was determined relative to PEG/PEO standards using gel permeation chromatography (Tosoh HLC-8420 GPC) equipped with an RI detector at the Korea Polymer Testing and Research Institute (KOPTRI, Seoul, Republic of Korea).

### 3.5. Cell Culture

Human keratinocytes (HaCaTs), human dermal fibroblasts (HDFs), and murine melanoma cells (B16F10) were obtained from the American Type Culture Collection (ATCC, Rockville, MD, USA). The cells were cultured in Dulbecco’s modified Eagle’s medium (DMEM) with 10% fetal bovine serum (FBS) and 1% penicillin/streptomycin (P/S) at 37 °C in a 5% CO_2_ humidified atmosphere.

### 3.6. Cell Viability

Cell viability was assessed using a 3-(4,5-dimethylthiazol-2-yl)-2,5-diphenyltetrazolium bromide (MTT) assay [33]. Cells were seeded in a 96-well plate at a density of 1 × 10^4^ cells/well and incubated overnight. The cells were treated with different concentrations of the extracted samples for 1 h, followed by treatment with or without hydrogen peroxide (H_2_O_2_, 1 mM) for 24 h at 37 °C in a 5% CO_2_ humidified atmosphere. After incubation, the cells were treated with MTT solution (0.5 mg/mL) for 2 h. The medium was aspirated, DMSO was added to dissolve the formazan crystals, and absorbance was measured at 570 nm.

### 3.7. UVB Irradiation

Cells were seeded in a 6-well plate at 3 × 10^5^ cells/well and incubated at 37 °C in a 5% CO_2_ incubator overnight. The cultured cells were starved in serum-free DMEM for 24 h and treated with extracted samples for 1 h. Then, the cells were rinsed with phosphate-buffered saline (PBS) and irradiated with UVB with a thin PBS layer. UVB irradiation was performed using a Bio-link Crosslinker BLX-312 (Vilber Lourmat, Collégien, France) at a peak emission wavelength of 312 nm. After UVB irradiation, the cells were cultured for 24 h in serum-free DMEM containing various concentrations of the samples.

### 3.8. Real-Time PCR (qPCR)

Total RNA was extracted from the cultured cells using an EcoPURE total RNA Kit (EcoTech Biotechnology, Erzurum, Turkey). The isolated RNA was synthesized into cDNA using the AmfiRivert cDNA Synthesis Platinum Master Mix (GenDEPOT, Katy, TX, USA). Matrix metaloproteinase-1 (MMP-1) mRNA expression levels were analyzed using real-time PCR with gene-specific primers. Primer sequences used were as follows: MMP-1 (Forward; CCCCAAAAGCGTGTGACA, Reverse; GGTAGAAGGGATTTGTGCG), GAPDH (Forward; GTCTCCTCTGACTTCAACAGCG, Reverse; ACCACCCTGTTGCTGTAGCCAA). RT-PCR was conducted using the Step One Plus Real-Time PCR system (Thermo Fisher Scientific, Waltham, MA, USA) with AccuPOWER 2× Green Star qPCR Master Mix (Bioneer, Daejeon, Republic of Korea).

### 3.9. Western Blot

Cultured cells were lysed in radioimmunoprecipitation assay (RIPA) lysis buffer containing a protease and phosphatase inhibitor cocktail. The protein concentration was evaluated using the BCA assay and heated at 95 °C for 5 min. The proteins were separated using 10% SDS acrylamide gels, and the separated proteins were transferred onto polyvinylidene fluoride (PVDF) membranes. The membrane was blocked with EveryBlot Blocking buffer at room temperature for 15 min. The blocked membrane was incubated with primary antibodies (MMP-1; sc58377, β-actin; 4970s, TRP-1; ab1708676, TRP-2; ab74073 and tyrosinase; ab170905) at 4 °C for overnight. The membrane was then rinsed with TBST and incubated with secondary antibodies (Anti-mouse IgG, HRP-linked antibody; 7076s and Anti-rabbit IgG, HRP-linked antibody; 7074s) at room temperature for 1 h. Protein expression was visualized using Fusion FX (Vilber Lourmat, Marne-la-Vallée, France) at Korea Basic Science Instititue (KBSI) Gwangju center.

### 3.10. Melanin Content

B16F10 cells (murine melanoma) were seeded in a 60 mm cell culture plate at 1 × 10^5^ cells/plate and incubated at 37 °C in a 5% CO_2_ incubator overnight. Cells were pretreated with samples for 1 h, followed by treatment with α-melanocyte-stimulating hormone (α-MSH, 100 nM) for 72 h. Then, the cultured medium was harvested, and the absorbance was measured at 490 nm.

### 3.11. Cellular Tyrosinase Assay

B16F10 cells were seeded at a density of 1 × 10^5^ cells/plate in a 60 mm plate. After 24 h of incubation, the cells were treated with samples for 1 h and with α-MSH (100 nM) for 72 h. Then, the cells were lysed with RIPA lysis buffer and centrifuged at 10,000× *g* for 10 min. The supernatant was collected, and the protein content was quantified using the BCA assay. Then, 40 μL (protein, 30 μg) of the supernatant and 100 μL of L-DOPA (3,4-dihydroxyphenylalanine, 10 mM) were mixed in a 96-well plate and incubated for 1 h at 37 °C. The absorbance was measured at 490 nm.

### 3.12. Statistical Analysis

All of the experiments were conducted in triplicate, and the data are expressed as the mean ± standard deviation. Normality tests were performed to determine if the data followed a normal distribution. For normally distributed data, statistical significance was evaluated using Student’s *t*-test with the SPSS program (Chicago, IL, USA), and significant differences were reported as *p* < 0.05. Pearson’s correlation analysis was performed using SPSS, with the significance level set at *p* < 0.01.

## 4. Conclusions

Ultrasound and enzymatic treatments have been employed to produce eco-friendly collagen peptides in alignment with sustainable development and blue growth strategies. Low-molecular-weight collagen peptides have demonstrated potential as materials with anti-oxidant, anti-wrinkle, and whitening properties on cultured skin cell lines. However, further research, such as 3D cell studies, animal studies, and human skin tissue experiments, is needed to demonstrate more definitive anti-wrinkle and whitening effects. This study has simplified the manufacturing process and achieved a significant yield of low-molecular-weight peptides through ultrasound and enzyme treatments. These advancements are expected to enhance the industrial application of collagen peptides.

## Figures and Tables

**Table 1:** Molecular weight analysis of UEs.

	UE	UEF1	UEF2
Number Average Molecular weight (Mn, Da)	619	650	360
Weight Average Molecular weight (Mw, Da)	1095	1100	468
Z Average Molecular weight (Mz, Da)	2824	2202	710

## Data Availability

Data contained within the article.
